# 1,2;5,6-Di-*O*-isopropyl­idene-3-*C*-nitro­methyl-α-d-allofuran­ose

**DOI:** 10.1107/S1600536811017314

**Published:** 2011-05-14

**Authors:** Qiurong Zhang, Yu Ke, Weiyan Cheng, Pengyun Li, Hongmin Liu

**Affiliations:** a New Drug Reseach & Development Center, Zhengzhou University, Zhengzhou 450001, People’s Republic of China

## Abstract

The mol­ecule of the title compound, C_13_H_21_NO_8_, consists of two methyl­enedi­oxy rings and one tetra­hydro­furan ring. In the crystal, inter­molecular O—H⋯O hydrogen bonds link the mol­ecules into helical chains running along the 6_1_ screw axis. Weak inter­molecular C—H⋯O hydrogen bonds help to stabilize the crystal packing. Voids of 245 Å^3^ per unit cell occur.

## Related literature

For details of the synthesis, see: Saito *et al.* (2002 ▶). For recent studies of the biological activity of aza­sugars, see: Loiseleur *et al.* (2007 ▶); Rahman *et al.* (2008 ▶).
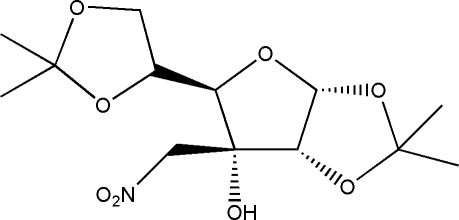


## Experimental

### 

#### Crystal data

C_13_H_21_NO_8_
*M* *_r_* = 319.31
Hexagonal, 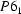

*a* = 13.2581 (19) Å
*c* = 16.462 (3) Å
*V* = 2506.0 (7) Å^3^
*Z* = 6
Mo *K*α radiation
μ = 0.11 mm^−1^
*T* = 291 K
0.24 × 0.20 × 0.20 mm

#### Data collection

Rigaku R-AXIS-IV diffractometer
Absorption correction: multi-scan (*ABSCOR*; Higashi, 1995 ▶) *T*_min_ = 0.975, *T*_max_ = 0.979
8380 measured reflections
1612 independent reflections
1534 reflections with *I* > 2σ(*I*)
*R*_int_ = 0.047

#### Refinement

*R*[*F* ^2^ > 2σ(*F* ^2^)] = 0.061
*wR*(*F* ^2^) = 0.164
*S* = 1.08
1612 reflections
205 parameters
1 restraint
H atoms treated by a mixture of independent and constrained refinement
Δρ_max_ = 0.43 e Å^−3^
Δρ_min_ = −0.26 e Å^−3^

### 

Data collection: *R-AXIS-IV Software* (Rigaku, 1997 ▶); cell refinement: *R-AXIS-IV Software*; data reduction: *R-AXIS-IV Software*; program(s) used to solve structure: *SHELXS97* (Sheldrick, 2008 ▶); program(s) used to refine structure: *SHELXL97* (Sheldrick, 2008 ▶); molecular graphics: *TEXSAN* (Molecular Structure Corporation, 1992 ▶); software used to prepare material for publication: *TEXSAN*.

## Supplementary Material

Crystal structure: contains datablocks I, global. DOI: 10.1107/S1600536811017314/cv5090sup1.cif

Structure factors: contains datablocks I. DOI: 10.1107/S1600536811017314/cv5090Isup2.hkl

Additional supplementary materials:  crystallographic information; 3D view; checkCIF report

## Figures and Tables

**Table 1:** Hydrogen-bond geometry (Å, °)

*D*—H⋯*A*	*D*—H	H⋯*A*	*D*⋯*A*	*D*—H⋯*A*
O3—H3*E*⋯O6^i^	0.90 (8)	1.95 (8)	2.814 (5)	161 (7)
C1—H1*A*⋯O3^ii^	0.98	2.37	3.258 (4)	151
C5—H5*A*⋯O1^iii^	0.98	2.50	3.320 (4)	141

Symmetry codes: (i) 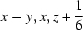; (ii) 
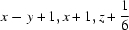
; (iii) 
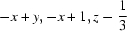
.

**Fig. 1.**
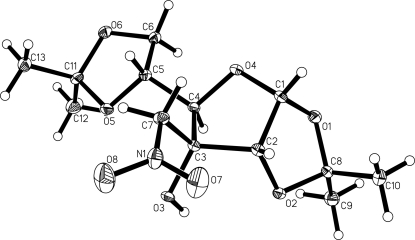
The molecular structure of (I) showing the atomic numbering and 30% probability displacement ellipsoids. H atoms omitted for clarity.

**Fig. 2.**
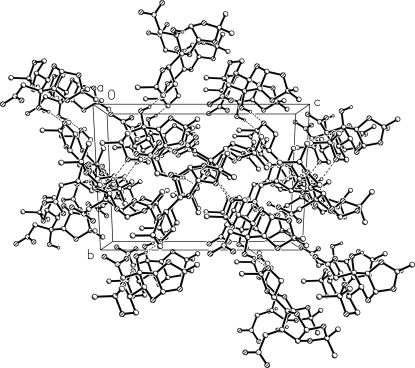
Packing diagram.

## supplementary crystallographic information

### Comment

Azasugars were recently used as novel glycosyls to synthesize novel *N*-nucleosides (Loiseleur *et al.*, 2007; Rahman *et al.*, 2008).  Herewith we report the synthesis and crystal structure of the title compound (I) prepared in enantiomerically pure form from 1,2;5,6-di-*O*-isopropylidene-3-carbonyl-α-*D*-glucofuranose (Saito *et al.*, 2002) at room tempeature, whose raw material was D-glucose.

The molecule of (I) consists of two methylenedioxy rings and one tetrahydrofuran ring (Fig. 1). In (I), the tetrahydrofuran ring fuses with one methylenedioxy ring, having the *cis* arrangement at the ring junctions and giving a V-shaped molecule. The angles O1—C8—O2, O5—C11—O6, C9—C8—C10 and C12—C11—C13 around the two isopropylidenes are 104.1 (4), 105.5 (4), 113.9 (5) and 114.2 (6)°, respectively.

In the crystal structure, intermolecular O—H···O hydrogen bonds (Table 1) link the molecules into helical chains running along screw axis 6_1_, and weak intermolecular C—H···O hydrogen bonds (Table 1) help to stabilize the crystal packing.

### Experimental

All reagents and solvents were used as obtained without further purification. 1,2;5,6-di-*O*-isopropylidene-3-*C*-(nitromethyl)- α-*D*-allofuranose was synthesized from 1,2;5,6-di-*O*-isopropylidene-3-carbonyl-α-*D*-glucofuranose as described previously by Saito *et al.* (2002), whose starting material was D-glucose. To a solution of 1,2;5,6-di-*O*-isopropylidene-3-carbonyl -α-*D*-Glucofuranose (7.0 g, 27 mmol) in tetrahydrofuran (50 ml) was added CH_3_NO_2_ (10.5 ml) and potassium fluoride (3.0 g).The mixture was stirred at room temperature for 6 h. The reaction mixture was then concentrated *in vacuo* and extracted with water and EtOAc, dried (Na_2_SO_4_), and evaporated. The residue was recrystalied in CH_3_OH to yield the title compound. Crystals suitable for X-ray analysis were grown by slow evaporation from methanol at room temperature for two weeks. *R*_f_ = 0.7 (CHCl_3_/EtOAc, 7:3); mp: 110–111°C, [α]^20^_D_ = +96° (c, 1.0, CH_3_OH); ^1^H NMR (400 MHz, CDCl_3_) σ: 5.85(1*H*, d, *J* = 3.6 Hz), 4.97 (1*H*, d, *J* = 12 Hz), 4.89 (1*H*, d, *J* = 3.6 Hz), 4.49 (1*H*, d, *J* = 12 Hz), 4.13 (1*H*, m), 4.01(1*H*,m), 3.95 (1*H*, m), 3.89 (1*H*, d, *J* = 8.8), 3.27 (1*H*, s), 1.61 (3H, s), 1.47 (3*H*, s), 1.39 (3*H*, s), 1.36 (3*H*, s); ^13^C NMR (100 MHz, CDCl_3_) σ: 113.3, 110.4, 103.7, 81.7, 79.8, 78.5, 77.6, 72.9, 67.9, 26.6, 26.5, 26.5, 25.0.

### Refinement

Atom H3E was located on a difference map and isotropically refined. C-bound H atoms were placed geometrically and treated as riding on their parent atoms with C—H are 0.96 Å (methylene) or 0.93 Å (aromatic), and *U*_iso_(H) =1.2*U*_eq_(C). In the absence of any significant anomalous scatterers in the molecule, attempts to confirm the absolute structure by refinement of the Flack parameter in the presence of 1468 sets of Friedel equivalents led to an inconclusive value of 10 (10). Therefore, the Friedel pairs were merged before the final refinement and the absolute configuration was assigned to correspond with that of the known chiral centres in a precursor molecule, which remained unchanged during the synthesis of the title compound. The porous crystal packing exhibits voids of 245 Å^3^.

### Crystal data

**Table:**

C_13_H_21_NO_8_	*D*_x_ = 1.270 Mg m^−^^3^
*M**_r_* = 319.31	Melting point = 383–384 K
Hexagonal, *P*6_1_	Mo *K*α radiation, λ = 0.71073 Å
Hall symbol: P 61	Cell parameters from 398 reflections
*a* = 13.2581 (19) Å	θ = 2–25.1°
*c* = 16.462 (3) Å	µ = 0.11 mm^−^^1^
*V* = 2506.0 (7) Å^3^	*T* = 291 K
*Z* = 6	Prismatic, colourless
*F*(000) = 1020	0.24 × 0.20 × 0.20 mm

### Data collection

**Table:**

Rigaku R-AXIS-IV diffractometer	1612 independent reflections
Radiation source: fine-focus sealed tube	1534 reflections with *I* > 2σ(*I*)
graphite	*R*_int_ = 0.047
Detector resolution: 0 pixels mm^-1^	θ_max_ = 25.5°, θ_min_ = 1.8°
Oscillation frames scans	*h* = −13→16
Absorption correction: multi-scan (*ABSCOR*; Higashi, 1995)	*k* = −16→0
*T*_min_ = 0.975, *T*_max_ = 0.979	*l* = −19→19
8380 measured reflections	

### Refinement

**Table:**

Refinement on *F*^2^	Secondary atom site location: difference Fourier map
Least-squares matrix: full	Hydrogen site location: inferred from neighbouring sites
*R*[*F*^2^ > 2σ(*F*^2^)] = 0.061	H atoms treated by a mixture of independent and constrained refinement
*wR*(*F*^2^) = 0.164	*w* = 1/[σ^2^(*F*_o_^2^) + (0.0962*P*)^2^ + 1.0744*P*] where *P* = (*F*_o_^2^ + 2*F*_c_^2^)/3
*S* = 1.08	(Δ/σ)_max_ < 0.001
1612 reflections	Δρ_max_ = 0.43 e Å^−^^3^
205 parameters	Δρ_min_ = −0.26 e Å^−^^3^
1 restraint	Extinction correction: *SHELXL97* (Sheldrick, 2008), Fc^*^=kFc[1+0.001xFc^2^λ^3^/sin(2θ)]^-1/4^
Primary atom site location: structure-invariant direct methods	Extinction coefficient: 0.008 (2)

### Special details

**Table:**

Geometry. All e.s.d.'s (except the e.s.d. in the dihedral angle between two l.s. planes) are estimated using the full covariance matrix. The cell e.s.d.'s are taken into account individually in the estimation of e.s.d.'s in distances, angles and torsion angles; correlations between e.s.d.'s in cell parameters are only used when they are defined by crystal symmetry. An approximate (isotropic) treatment of cell e.s.d.'s is used for estimating e.s.d.'s involving l.s. planes.
Refinement. Refinement of *F*^2^ against ALL reflections. The weighted *R*-factor *wR* and goodness of fit *S* are based on *F*^2^, conventional *R*-factors *R* are based on *F*, with *F* set to zero for negative *F*^2^. The threshold expression of *F*^2^ > σ(*F*^2^) is used only for calculating *R*-factors(gt) *etc*. and is not relevant to the choice of reflections for refinement. *R*-factors based on *F*^2^ are statistically about twice as large as those based on *F*, and *R*- factors based on ALL data will be even larger.

### Fractional atomic coordinates and isotropic or equivalent isotropic displacement parameters (Å^2^)

**Table:**

	*x*	*y*	*z*	*U*_iso_*/*U*_eq_	
O1	0.2338 (3)	0.7097 (4)	0.2334 (2)	0.0565 (10)	
O2	0.0552 (3)	0.6458 (3)	0.1793 (2)	0.0427 (8)	
O3	0.0092 (2)	0.5408 (3)	0.0319 (2)	0.0405 (7)	
O4	0.3029 (3)	0.6935 (3)	0.1082 (2)	0.0490 (9)	
O5	0.1802 (3)	0.4394 (3)	−0.0205 (3)	0.0597 (11)	
O6	0.3601 (3)	0.4537 (4)	−0.0197 (3)	0.0557 (9)	
O7	0.0437 (7)	0.7865 (7)	−0.0252 (4)	0.127 (3)	
O8	0.0234 (7)	0.6912 (7)	−0.1261 (4)	0.131 (3)	
N1	0.0764 (5)	0.7349 (5)	−0.0646 (3)	0.0639 (13)	
C1	0.2559 (4)	0.7508 (4)	0.1523 (3)	0.0472 (12)	
H1A	0.3068	0.8355	0.1502	0.057*	
C2	0.1347 (4)	0.7135 (4)	0.1170 (3)	0.0417 (10)	
H2A	0.1263	0.7800	0.1005	0.050*	
C3	0.1227 (4)	0.6337 (4)	0.0451 (3)	0.0358 (9)	
C4	0.2095 (3)	0.5932 (4)	0.0705 (3)	0.0362 (10)	
H4A	0.1721	0.5317	0.1114	0.043*	
C5	0.2641 (4)	0.5535 (5)	0.0066 (3)	0.0445 (11)	
H5A	0.2918	0.6081	−0.0392	0.053*	
C6	0.3605 (5)	0.5349 (5)	0.0379 (3)	0.0514 (12)	
H6A	0.4347	0.6070	0.0381	0.062*	
H6B	0.3438	0.5025	0.0923	0.062*	
C7	0.1610 (4)	0.7012 (5)	−0.0346 (3)	0.0496 (12)	
H7A	0.2360	0.7709	−0.0268	0.059*	
H7B	0.1703	0.6537	−0.0754	0.059*	
C8	0.1192 (4)	0.6842 (5)	0.2544 (3)	0.0463 (12)	
C9	0.0671 (6)	0.5833 (6)	0.3133 (4)	0.0650 (15)	
H9A	0.0672	0.5174	0.2892	0.097*	
H9B	−0.0114	0.5635	0.3258	0.097*	
H9C	0.1125	0.6050	0.3623	0.097*	
C10	0.1218 (6)	0.7930 (6)	0.2860 (4)	0.0693 (17)	
H10A	0.1542	0.8528	0.2453	0.104*	
H10B	0.1689	0.8196	0.3341	0.104*	
H10C	0.0440	0.7755	0.2986	0.104*	
C11	0.2428 (4)	0.3839 (5)	−0.0501 (4)	0.0552 (13)	
C12	0.1872 (7)	0.2647 (6)	−0.0156 (6)	0.093 (2)	
H12A	0.1886	0.2690	0.0426	0.139*	
H12B	0.2291	0.2269	−0.0332	0.139*	
H12C	0.1080	0.2210	−0.0340	0.139*	
C13	0.2482 (6)	0.3881 (7)	−0.1415 (4)	0.0729 (19)	
H13A	0.2860	0.4677	−0.1592	0.109*	
H13B	0.1707	0.3469	−0.1632	0.109*	
H13C	0.2915	0.3523	−0.1602	0.109*	
H3E	−0.017 (6)	0.497 (6)	0.077 (5)	0.067 (19)*	

### Atomic displacement parameters (Å^2^)

**Table:**

	*U*^11^	*U*^22^	*U*^33^	*U*^12^	*U*^13^	*U*^23^
O1	0.0408 (19)	0.077 (3)	0.0453 (19)	0.0249 (17)	−0.0092 (15)	−0.0111 (18)
O2	0.0312 (16)	0.0491 (19)	0.0426 (17)	0.0161 (15)	−0.0026 (13)	−0.0029 (14)
O3	0.0244 (15)	0.0419 (17)	0.0460 (17)	0.0098 (14)	−0.0045 (13)	0.0004 (15)
O4	0.0269 (16)	0.057 (2)	0.054 (2)	0.0135 (14)	−0.0040 (14)	−0.0109 (16)
O5	0.0323 (17)	0.066 (2)	0.075 (2)	0.0197 (16)	−0.0017 (17)	−0.028 (2)
O6	0.045 (2)	0.071 (2)	0.059 (2)	0.0357 (19)	0.0046 (17)	−0.0059 (19)
O7	0.193 (8)	0.185 (7)	0.089 (4)	0.159 (7)	−0.029 (4)	−0.013 (5)
O8	0.173 (6)	0.184 (7)	0.094 (4)	0.132 (6)	−0.057 (5)	−0.031 (5)
N1	0.095 (4)	0.069 (3)	0.044 (3)	0.053 (3)	−0.006 (3)	0.003 (2)
C1	0.033 (2)	0.041 (3)	0.056 (3)	0.010 (2)	−0.005 (2)	−0.010 (2)
C2	0.037 (2)	0.037 (2)	0.047 (3)	0.016 (2)	−0.003 (2)	0.002 (2)
C3	0.029 (2)	0.034 (2)	0.040 (2)	0.0126 (18)	−0.0018 (17)	0.0001 (18)
C4	0.026 (2)	0.040 (2)	0.035 (2)	0.0113 (18)	−0.0010 (17)	0.0023 (18)
C5	0.034 (2)	0.054 (3)	0.043 (2)	0.020 (2)	0.0006 (19)	−0.002 (2)
C6	0.044 (3)	0.067 (3)	0.047 (3)	0.031 (3)	0.000 (2)	−0.008 (3)
C7	0.041 (3)	0.058 (3)	0.046 (3)	0.022 (2)	0.003 (2)	0.011 (2)
C8	0.035 (2)	0.055 (3)	0.043 (3)	0.018 (2)	−0.0061 (19)	−0.011 (2)
C9	0.063 (4)	0.077 (4)	0.050 (3)	0.031 (3)	−0.006 (3)	0.003 (3)
C10	0.067 (4)	0.070 (4)	0.067 (4)	0.031 (3)	0.004 (3)	−0.019 (3)
C11	0.044 (3)	0.064 (3)	0.059 (3)	0.027 (3)	0.012 (2)	−0.010 (3)
C12	0.093 (5)	0.065 (4)	0.106 (6)	0.028 (4)	0.033 (5)	0.000 (4)
C13	0.060 (3)	0.105 (5)	0.061 (3)	0.047 (4)	−0.005 (3)	−0.023 (4)

### Geometric parameters (Å, °)

**Table:**

O1—C1	1.415 (7)	C5—C6	1.509 (7)
O1—C8	1.424 (6)	C5—H5A	0.9800
O2—C2	1.421 (6)	C6—H6A	0.9700
O2—C8	1.441 (6)	C6—H6B	0.9700
O3—C3	1.405 (5)	C7—H7A	0.9700
O3—H3E	0.90 (8)	C7—H7B	0.9700
O4—C1	1.402 (6)	C8—C9	1.510 (8)
O4—C4	1.428 (5)	C8—C10	1.517 (8)
O5—C5	1.429 (6)	C9—H9A	0.9600
O5—C11	1.441 (6)	C9—H9B	0.9600
O6—C6	1.432 (6)	C9—H9C	0.9600
O6—C11	1.445 (7)	C10—H10A	0.9600
O7—N1	1.171 (8)	C10—H10B	0.9600
O8—N1	1.204 (8)	C10—H10C	0.9600
N1—C7	1.484 (7)	C11—C12	1.483 (10)
C1—C2	1.540 (7)	C11—C13	1.506 (9)
C1—H1A	0.9800	C12—H12A	0.9600
C2—C3	1.542 (7)	C12—H12B	0.9600
C2—H2A	0.9800	C12—H12C	0.9600
C3—C7	1.525 (7)	C13—H13A	0.9600
C3—C4	1.551 (6)	C13—H13B	0.9600
C4—C5	1.513 (6)	C13—H13C	0.9600
C4—H4A	0.9800		
			
C1—O1—C8	108.2 (4)	H6A—C6—H6B	109.2
C2—O2—C8	106.1 (3)	N1—C7—C3	112.5 (4)
C3—O3—H3E	110 (4)	N1—C7—H7A	109.1
C1—O4—C4	108.4 (3)	C3—C7—H7A	109.1
C5—O5—C11	107.7 (4)	N1—C7—H7B	109.1
C6—O6—C11	108.0 (4)	C3—C7—H7B	109.1
O7—N1—O8	116.7 (7)	H7A—C7—H7B	107.8
O7—N1—C7	123.3 (5)	O1—C8—O2	104.1 (4)
O8—N1—C7	118.8 (6)	O1—C8—C9	109.1 (4)
O4—C1—O1	110.1 (4)	O2—C8—C9	108.1 (4)
O4—C1—C2	107.8 (4)	O1—C8—C10	110.0 (4)
O1—C1—C2	104.5 (4)	O2—C8—C10	111.1 (5)
O4—C1—H1A	111.4	C9—C8—C10	113.9 (5)
O1—C1—H1A	111.4	C8—C9—H9A	109.5
C2—C1—H1A	111.4	C8—C9—H9B	109.5
O2—C2—C1	104.8 (4)	H9A—C9—H9B	109.5
O2—C2—C3	109.3 (4)	C8—C9—H9C	109.5
C1—C2—C3	104.0 (4)	H9A—C9—H9C	109.5
O2—C2—H2A	112.7	H9B—C9—H9C	109.5
C1—C2—H2A	112.7	C8—C10—H10A	109.5
C3—C2—H2A	112.7	C8—C10—H10B	109.5
O3—C3—C7	106.1 (4)	H10A—C10—H10B	109.5
O3—C3—C2	114.6 (4)	C8—C10—H10C	109.5
C7—C3—C2	111.6 (4)	H10A—C10—H10C	109.5
O3—C3—C4	113.2 (3)	H10B—C10—H10C	109.5
C7—C3—C4	110.4 (4)	O5—C11—O6	105.5 (4)
C2—C3—C4	101.0 (3)	O5—C11—C12	108.0 (5)
O4—C4—C5	106.5 (3)	O6—C11—C12	110.6 (6)
O4—C4—C3	104.1 (3)	O5—C11—C13	110.4 (6)
C5—C4—C3	119.9 (4)	O6—C11—C13	107.8 (5)
O4—C4—H4A	108.6	C12—C11—C13	114.2 (6)
C5—C4—H4A	108.6	C11—C12—H12A	109.5
C3—C4—H4A	108.6	C11—C12—H12B	109.5
O5—C5—C6	102.0 (4)	H12A—C12—H12B	109.5
O5—C5—C4	109.4 (4)	C11—C12—H12C	109.5
C6—C5—C4	114.2 (4)	H12A—C12—H12C	109.5
O5—C5—H5A	110.3	H12B—C12—H12C	109.5
C6—C5—H5A	110.3	C11—C13—H13A	109.5
C4—C5—H5A	110.3	C11—C13—H13B	109.5
O6—C6—C5	102.2 (4)	H13A—C13—H13B	109.5
O6—C6—H6A	111.3	C11—C13—H13C	109.5
C5—C6—H6A	111.3	H13A—C13—H13C	109.5
O6—C6—H6B	111.3	H13B—C13—H13C	109.5
C5—C6—H6B	111.3		
			
C4—O4—C1—O1	91.7 (4)	C11—O5—C5—C4	−154.2 (4)
C4—O4—C1—C2	−21.7 (5)	O4—C4—C5—O5	166.8 (4)
C8—O1—C1—O4	−132.3 (4)	C3—C4—C5—O5	−75.7 (5)
C8—O1—C1—C2	−16.8 (5)	O4—C4—C5—C6	53.2 (5)
C8—O2—C2—C1	24.8 (5)	C3—C4—C5—C6	170.8 (4)
C8—O2—C2—C3	135.8 (4)	C11—O6—C6—C5	−29.7 (6)
O4—C1—C2—O2	111.9 (4)	O5—C5—C6—O6	37.9 (5)
O1—C1—C2—O2	−5.2 (5)	C4—C5—C6—O6	155.7 (4)
O4—C1—C2—C3	−2.9 (5)	O7—N1—C7—C3	53.9 (9)
O1—C1—C2—C3	−119.9 (4)	O8—N1—C7—C3	−112.8 (7)
O2—C2—C3—O3	34.0 (5)	O3—C3—C7—N1	53.1 (6)
C1—C2—C3—O3	145.5 (4)	C2—C3—C7—N1	−72.5 (5)
O2—C2—C3—C7	154.7 (4)	C4—C3—C7—N1	176.1 (4)
C1—C2—C3—C7	−93.8 (5)	C1—O1—C8—O2	32.4 (5)
O2—C2—C3—C4	−88.0 (4)	C1—O1—C8—C9	147.7 (4)
C1—C2—C3—C4	23.5 (4)	C1—O1—C8—C10	−86.7 (5)
C1—O4—C4—C5	164.9 (4)	C2—O2—C8—O1	−35.5 (5)
C1—O4—C4—C3	37.3 (5)	C2—O2—C8—C9	−151.4 (4)
O3—C3—C4—O4	−159.9 (4)	C2—O2—C8—C10	82.9 (5)
C7—C3—C4—O4	81.3 (4)	C5—O5—C11—O6	15.2 (6)
C2—C3—C4—O4	−36.8 (4)	C5—O5—C11—C12	133.6 (6)
O3—C3—C4—C5	81.4 (5)	C5—O5—C11—C13	−100.9 (5)
C7—C3—C4—C5	−37.4 (5)	C6—O6—C11—O5	10.1 (6)
C2—C3—C4—C5	−155.6 (4)	C6—O6—C11—C12	−106.5 (6)
C11—O5—C5—C6	−32.9 (5)	C6—O6—C11—C13	128.0 (5)

### Hydrogen-bond geometry (Å, °)

**Table:**

*D*—H···*A*	*D*—H	H···*A*	*D*···*A*	*D*—H···*A*
O3—H3E···O6^i^	0.90 (8)	1.95 (8)	2.814 (5)	161 (7)
C1—H1A···O3^ii^	0.98	2.37	3.258 (4)	151.
C5—H5A···O1^iii^	0.98	2.50	3.320 (4)	141.

Symmetry codes: (i) *x*−*y*, *x*, *z*+1/6; (ii) *x*−*y*+1, *x*+1, *z*+1/6; (iii) −*x*+*y*, −*x*+1, *z*−1/3.
